# Exploring US patient and caregiver perspectives on burden associated with Friedreich Ataxia

**DOI:** 10.1186/s41687-026-01067-4

**Published:** 2026-04-18

**Authors:** Richard Lawson, Shobhana Natarajan, Julia R. Correll, Amy Clark, Mona L. Martin, Donald M. Bushnell, Juliana Setyawan

**Affiliations:** 1 Biogen, 225 Binney St, Cambridge, MA 02142 USA; 2 Thermo Fisher Scientific, PPD Evidera Patient Centered Research, Wilmington, NC USA; 3 Thermo Fisher Scientific, PPD Evidera Patient Centered Research, London, UK; 4 Formerly Biogen, 225 Binney St, Cambridge, MA 02142 USA

**Keywords:** Friedreich Ataxia, Patient experience, Patient perceived burden, Quality of life

## Abstract

**Background:**

Friedreich ataxia (FA) is a hereditary degenerative disease with clinical manifestations in multiple organs characterized by progressive gait and limb ataxia. Qualitative interviews were conducted with patients and caregivers to characterize the burden experienced related to FA symptoms and impacts.

**Methodology:**

A sample of 20 FA patients and 11 caregivers were recruited from the Friedreich Ataxia Research Alliance (FARA) registry and FA Facebook communities. Individual qualitative interviews were conducted via telephone by trained qualitative research interviewers to last no more than 45 min. Audio files were transcribed and coded to group concepts by similarity of theme or concept using ATLAS.ti software.

**Results:**

The 20 patients interviewed ranged from 22 to 36 years of age (mean 28.45), 75% were female and seven (35%) were still working full-time. The participants interviewed required an average of 55.5 h per week of assistance from a caregiver. The most frequently reported symptoms were neurological including impaired speech (*n* = 14; 70%), loss of balance (*n* = 10; 50%) and decreased muscle coordination (*n* = 8; 40%). Negative impacts of symptoms included limited function in activities of daily living such as walking (n=15; 75%), dressing (n=11; 55%), limited social activities (n=11; 55%), personal care and hygiene (n=8; 40%), and falls (n=6; 30%). Additionally, 91% of caregivers reported observing falls, and 73% reported issues with patient personal care and hygiene. Patients rated their symptom bothersomeness at 6.7, impact of FA on their ability to live their daily lives at 7.1, and their financial burden due to FA at 6.5, all on a 9-point scale where higher scores indicate worse outcomes.

**Conclusions:**

Results show that from both the patient and caregiver perspective living with FA is highly burdensome for patients, negatively impacting their daily lives, and limiting their ability to lead a functional life, in areas like mobility, self-care, and productivity.

## Background

Friedreich ataxia (FA) is a hereditary, autosomal recessive, degenerative disease characterized by progressive gait and limb ataxia, with clinical manifestations in multiple organs [1]. In most cases, FA is caused by an intronic GAA triplet repeat expansion in the FXN gene resulting in decreased frataxin—a mitochondrial protein. Evidence suggests that FA is also a result of iron accumulation in mitochondria, which leads to over production of free radicals, consequently resulting in cellular death and damage [2]. FA affects approximately one in 50,000 to 100,000 individuals in the United States (US), and typically manifests between the ages of five and 20 years [1]. Gait instability and limb ataxia are the most common manifestations in those with FA. Other neurological signs/symptoms include an absence of reflexes, loss of vibration sense and proprioception, dysarthria, hearing loss, vision impairment, musculoskeletal abnormalities, nystagmus, and mild olfactory dysfunction [1, 3]. Non-neurological comorbidities include scoliosis, hypertrophic cardiomyopathy, and diabetes mellitus [4].

FA is a complex disease requiring a multidisciplinary approach to management and often causes a significant burden on formal and informal caregivers. In the US, healthcare costs are significantly higher in advanced stages of disease. Preliminary information has been gathered describing the day-to-day challenges for people with ataxia and their relatives and friends [5].

In early 2023, the first and only drug treating FA was FDA-approved and expanded to the European Union (EU) and Switzerland in 2024 [6]. Patient-reported outcome (PRO) measures addressing FA are limited. Those with published support include the Friedreich’s Ataxia-Health Index (FA-HI) [7] and the Friedreich’s Ataxia – Activities of Daily Living (FA-ADL) [8]. This study further describes the patient, and caregiver experiences living with FA, and relates these results to scores from several objective measures that were administered alongside interviews.

## Methods

### Study design and target sample

This was a non-interventional study, aiming to capture the holistic burden of illness across multiple domains from the perspective of adult patients with FA and caregivers of patients with FA, including aspects of disease- and treatment-related burden, financial burden, and the overall humanistic burden of FA.

The targeted sample included adult patients with FA and caregivers of patients with FA across the US, who were recruited from the Friedreich’s Ataxia Research Alliance (FARA) registry and FA Facebook communities. None of the caregivers interviewed provided care to an interviewed patient; thus, these were each independent interviews.

### Study measures

Data collection included: (1) Self-administered, web-enabled instruments; a screener; and a pre-interview survey; and (2) Qualitative online interviews. The exploratory qualitative interviews were designed to capture deeper insights, narrative, and explanations about their experiences and descriptions of areas which constituted disease related burden. All interviews were conducted in English by trained interviewers from a market research company and lasted 30 to 45 min each. Audio files were transcribed for analysis.

Descriptive data captured during patient enrollment included sociodemographic and clinical characteristics and patient responses to the EQ-5D-3 L, WPAI-SHP, and Friedreich Ataxia – FA-ADL questionnaires. Descriptive data captured during caregiver enrollment included sociodemographic characteristics and caregiver responses to the WPAI-SHP (in relation to caring for the person with FA). These data were transferred with a data dictionary to the analysis team along with the interview transcripts.

The interview guides were developed using existing clinical literature. Patient and caregiver interview guides explored current and past FA treatment, FA-related symptoms, impact of symptoms on quality of life (QOL) and daily functional outcomes, financial burden, and FA-related healthcare resource utilization and where relevant the role of the caregiver.

During the interviews, patients and caregivers were asked to provide three ratings: (1) how bothersome their / their child’s symptoms were to them in their daily lives, using a numeric rating scale (NRS) of 1 to 9 (1 = not bothersome; 9 = extremely bothersome); (2) how their / their child’s FA impacted day-to-day lives on an NRS of 1 to 9 (1 = no impact at all; 9 = extremely high impact; and 3) current financial burden associated with FA on an NRS of 1 to 9 (1 = no burden at all; 9 = extremely high burden).

The interviewed patients had a range of bulbar and speech function and interviewers ensured that questions and responses were well understood. A few patients had their caregiver present on the call to support on this, but these responses were from the patient and not caregiver perspective. Interviews were capped at 45 min to support patient durability, which resulted in some questions not being asked to all patients.

### Data management and analysis

A qualitative analysis plan (QAP) was developed, which outlined the study objectives, the planned qualitative and descriptive analyses, and shell tables for presenting interview results. Interview transcripts were coded using ATLAS.ti to identify thematic concepts [9]. The primary goal of transcript coding was to organize and catalog participant descriptions of their FA symptoms and impacts on their daily lives and health-related QOL. A coding framework was developed to provide a roadmap for code assignment. The initial draft of the coding framework was based on the study objectives, interview guide content, and the QAP. The coding framework was revised as codes were identified in the transcripts. All transcript data were coded to allow similar concepts to be organized into groups, to look at the predominance of thematically related content from patient responses about their symptoms, impacts, and treatment experiences. Symptoms and impacts are summarized in the results tables in two ways; for each symptom/impact the following are displayed: (1) the number of expressions about each specific symptom/impact and the proportion of those expressions out of the total number of symptom or impact expressions, and (2) the number of patients or caregivers who reported each symptom/impact and the proportion of patients/caregivers out of the total.

All data was kept strictly confidential in accordance with local, state, and federal regulations and International Conference on Harmonization Good Clinical Practice guidelines. Data quality checks included the assessment of saturation of concept and inter-coder agreement (ICA).

Descriptive data from the pre-interview survey were analyzed, and tables were prepared describing patient and caregiver characteristics (N, mean, standard deviation, percent, as appropriate). The EQ-5D-3 L, WPAI-SHP, and FA-ADL were scored via the published scoring algorithms and used to develop descriptive tables. For select tables, this data was displayed beside qualitative responses from the interview session. The analysis for this study used a mixed methods approach, as qualitative data are presented alongside quantitative data to enhance interpretation of study results.

Evidera extracted severity and bothersomeness ratings from the interview transcripts and entered those data into an Excel file for tabulation and presentation in descriptive tables. Edit checks were used to ensure that entered data were valid (e.g., participant responses were within allowable ranges).

Depending on timing of interview, the amount of information the participant was providing, and limitations to participant stamina, not all interview guide questions were able to be covered in each interview. Results are presented as a percent of the number of participants who responded to each question. Therefore, the denominator is less than 20 in some cases (for patients) or less than 11 in some cases (for caregivers).

### Data quality

Saturation of concept (the point at which no further new information is being obtained from the interviews) was assessed using the total group of patient and caregiver interviews. The interviews were arranged chronologically by date of conduct and broken into 6 groups of 5 or 6 transcripts each. Following the coding, each subsequent group of transcripts was compared to the previous group to identify new concepts that arose in the coding process. Ideally, the final group of transcripts should have little to no new information forthcoming.

ICA was evaluated by independently dual coding three transcripts and comparing the coded results for consistency in code assignment. The results are reported as an averaged percent agreement, ideally showing over 90% agreement to represent consistency in the coding process [10].

## Results

### Data quality checks

Saturation of concept assessment showed 50% of all symptom and impact concepts appearing in the first transcript group, 11.0% appeared in the second group, 11.9% in the third group, 13.6% in the fourth group, 6.8% in the fifth group, and another 6.8% in the final transcript group, which is less than 2% higher than the recommended threshold of 5% for new information to still be forthcoming in exploratory qualitative research [11].

While it is possible that additional interviews may continue to bring up new concepts, it should be noted that the descriptions of these concepts are highly detailed and individual to the patient and caregiver’s preferred terminology. Many of them are also granular descriptions related to other concepts (e.g., difficulty sitting, difficulty getting to school, need for support at school). Therefore, saturation of concept results for this study were determined to be acceptable.

ICA analysis results showed the average agreement between coders for recognizing a concept needing to have a code assigned was 86.9%, and the average agreement on code assignment was 89.1%.

### Participant characteristics (description of sample)

The study sample consisted of 20 adult patients with FA and 11 caregivers of patients with FA. Table 1 presents patient and caregiver sociodemographic characteristics. The average age of patients with FA was 28.5 years old, and they were mostly female (*n* = 15/20, 75.0%). Employment status was mixed for those with FA, with 35.0% (*n* = 7) employed full-time, 30.0% (*n* = 6) disabled, and 20.0% (*n* = 4) employed part-time.

**Table 1:** Sociodemographic Descriptives for Patients and Caregivers (*n* = 31)

Characteristic	Patients *n*=20 *n* (%)	Caregivers *n*=11 *n* (%)
**Age**		
Mean (SD)	28.5 (4.62)	49.6 (8.4)
Range	[22–36]	[32–64]
**Age of person with FA they care for**		
Mean (SD)		20.4 (7.6)
Range		[12–38]
**Sex**		
Male	5 (25.0%)	3 (27.3%)
Female	15 (75.0%)	8 (72.7%)
**Employment status**		
Employed full-time	7 (35.0%)	9 (81.8%)
Employed part-time	4 (20.0%)	1 (9.1%)
Homemaker	1 (5.0%)	---
Disabled	6 (30.0%)	---
Retired	0 (0%)	1 (9.1%)
Full or part-time student	2 (10.0%)	---
Currently seeking employment or underemployment	3 (15.0%)	---
Never employed	2 (10.0%)	---
Prefer not to say	0 (0%)	---
**Age when diagnosed with FA**		
Mean (SD)	15.2 (6.40)	
Range	[5–27]	
**Whether currently receiving caregiver support**		
Yes	13 (65.0%)	
No	7 (35.0%)	
**Number of formal and informal caregivers**		
Formal	1.8 (2.71) [0–8]	
Informal	1.4 (0.77) [0–3]	

The average age for caregivers was 49.6 years old, and the average age of their child with FA was 20.4 years. Most caregivers were female and mothers (*n* = 8/11, 72.7%). Nearly two thirds of caregivers were married (*n* = 7/11, 63.6%), and most were employed full-time (*n* = 9; 81.8%). Caregivers reported having an average of 1.9 children, including 1.3 diagnosed with FA. All caregivers reported their child with FA had been reported at least one year ago.

All patients and caregivers lived in the US and were distributed geographically among Alabama, Arizona, California, Connecticut, Florida, Georgia, Iowa, Louisiana, Michigan, Minnesota, Missouri, New Hampshire, New Jersey, New York, North Carolina, Oklahoma, Pennsylvania, South Carolina, Texas, Washington, and West Virginia.

### Patient history with FA

The average diagnosis age of patients with FA was 15.2 years old. Caregivers described their children experiencing clumsiness, loss of balance, falls, and other medical comorbidities that led them to seek medical attention. Example quotations are as follows:

*I’ve always been clumsy*,* but it was more pronounced… [when] you close your eyes and stand up… I don’t think I can do that without closing onto something. So that prompted me to actually go to a neurologist.* (Patient 27/female)

… *the scoliosis and the hypertrophic cardiomyopathy were*,* like*,* the first two medical things that we were like*,* all right*,* we got to deal with this.* (CG of 13-year-old)

*We noticed that our oldest child was very clumsy and fell often… He just didn’t have control over his body…* (CG of 20-year-old)

*… when she was in first grade that she didn’t have the balance of a normal child. We could not get her to do anything athletic… the strength wasn’t there.* (CG of 12-year-old)

All caregivers (*n* = 11/11, 100.0%) found out that their children suffered from FA after seeking a genetic test based on the symptoms observed. Most patients (*n* = 12/13, 92.3%) and caregivers (*n* = 5/6, 83.3%) reported that the FA diagnosis was made by a neurologist. Over half of patients (*n* = 5/9; 55.6%) and one quarter of caregivers (*n* = 2/8; 25.0%) reported visiting “many” or at least four healthcare providers (HCPs) before receiving an FA diagnosis.

When asked how their diagnosis was explained to them, patients often noted that, since they were so young at diagnosis, the information was given to their parents, and they were not aware of it. Two caregivers (*n* = 2/8; 25.0%) reported researching the disease online before meeting with the doctor, so they were more prepared to ask questions and did not need as much explanation of the disease itself. Several caregivers reported negative experiences related to being told by the HCP about the FA diagnosis, including the HCP not explaining the disease or potential scenarios very well.

Most patients (*n* = 11/12; 91.7%) reported worsening of their symptoms over time, including a decline in walking, voice changes, and choking. Most caregivers (*n* = 7/8; 87.5%) observed a worsening in their child’s FA symptoms over time such as balance and walking.

### Symptom burden

During the interviews, patients were asked to describe the symptoms they experience that they associated with their FA. Caregivers were asked to report on the symptoms observed in the person with FA that they cared for.

Table 2 shows the predominance of symptom reports brought up during the interviews. The predominant language used to express concepts is shown (for example, most patients used the terms tiredness and fatigue), and how much symptoms were talked about compared to all other symptoms shows in total mentions column, while the proportion of the sample having the symptom shows is in the last column. Patients reported a broad symptom picture (22 symptoms in total) that contributed to their FA burden (Table 2). The symptoms most frequently reported by patients were neurological and included: impaired speech (*n* = 14/20; 70.0%), decrease of muscle coordination (*n* = 13/20; 65.0%), and loss of balance (*n* = 11/20; 55.0%). Other symptoms included fatigue (*n* = 9/20; 45.0%), development of cardiovascular and endocrinologic comorbidities (*n* = 9/20; 45.0%), scoliosis (*n* = 9/20; 45.0%), and hearing loss/issues (*n* = 6/20; 30.0%). All other symptoms were reported by four or fewer patients.

**Table 2:** Predominance of symptom expressions (Reported by persons with FA, *n* = 20)

Patient-Reported Symptoms of Friedreich’s Ataxia	Number Patient Language Expressions within Concept	% of Total Symptom Expressions (*n* = 189)	# Patients Contributing to Concept Expression (*n* = 20)	% of Patients Contributing (*n* = 20)
**Neurological symptoms**	**108**	**57%**		
Impaired Speech *slow or slurred*	26	13.8%	14	70.0%
Muscle Coordination Decrease	29	8.5%	13	65.0%
Loss of Balance	21	11.1%	11	55.0%
Hearing Loss/Issues	11	5.8%	6	30.0%
Muscle Weakness	5	2.6%	4	20.0%
Vision Loss/Issues	4	2.1%	4	20.0%
Difficulty Swallowing	4	2.1%	3	15.0%
Muscle Spasm/Cramps	3	1.6%	2	10.0%
Nerve Pain	4	2.1%	2	10.0%
Loss of Proprioception *sense of self-movement*,* force*,* and body position*	1	0.5%	1	5.0%
**Energy-related symptoms**	**35**	**19%**		
Fatigue	16	8.5%	9	45.0%
Tiredness	15	7.9%	8	40.0%
Exhaustion	2	1.1%	1	5.0%
Low Energy	2	1.1%	1	5.0%
**Genitourinary symptoms**	**13**	**7%**		
Urinary Incontinence	10	5.3%	3	15.0%
Urinary Urgency	3	1.6%	2	10.0%
**Other symptoms**	**33**	**17%**		
Development of Cardiovascular and Endocrinologic Comorbidities	17	9.0%	9	45.0%
Scoliosis *spine curvature*	10	5.3%	9	45.0%
General Pain/Discomfort	1	0.5%	1	5.0%
Leg Pain	3	1.6%	1	5.0%
Poor Circulation	1	0.5%	1	5.0%
Poor Posture	1	0.5%	1	5.0%

When asked to rate the bothersomeness of their FA symptoms overall from 1 (not bothersome) to 9 (extremely bothersome), patients reported a mean rating of 6.7 and a median of 7 (range 4 to 9). In assessing symptom bothersomeness and age of diagnosis we saw a strong correlation (*r*=-0.74) between increasing symptom bothersomeness and earlier age of diagnosis.

Like patients, caregivers described wide-ranging symptoms (33 in total) that often progressed over time (Table 3). The symptoms most frequently observed by caregivers were development of cardiovascular and endocrinologic comorbidities (*n* = 9/11; 81.8%) and scoliosis (*n* = 9/11; 81.8%), followed by the neurological symptoms of decrease of muscle coordination (*n* = 8/11; 72.7%), loss of balance (*n* = 7/11; 63.6%), muscle weakness (*n* = 5/11; 45.5%), impaired speech (*n* = 4/11; 36.4%), and muscle tone increase (*n* = 4/11; 36.4%). In addition, caregivers frequently reported energy-related symptoms of tiredness (*n* = 7/11; 63.6%) and fatigue (*n* = 6/11; 54.5%), as well as foot abnormalities (*n* = 4/11; 36.4%). All other symptoms were reported by only one or two caregivers. Overall, caregivers described a larger number of symptoms than patients.

**Table 3:** Symptom code summary table (Reported by caregivers of persons with FA, *n* = 11)

Caregiver-Reported Symptoms of Friedreich’s Ataxia	Number Caregiver Language Expressions within Concept	% of Total Symptom Expressions (*n* = 164)	# Caregivers Contributing to Concept Expression (*n* = 11)	% of Caregivers Contributing (*n* = 11)
**Neurological symptoms**	**76**	**46%**		
Muscle Coordination Decrease	15	6.1%	8	72.7%
Loss of Balance	18	11.0%	7	63.6%
Muscle Weakness	8	4.9%	5	45.5%
Impaired Speech *slow or slurred*	8	4.9%	4	36.4%
Muscle Tone Increase	4	2.4%	4	36.4%
Difficulty Swallowing	2	1.2%	2	18.2%
Loss of Reflexes	2	1.2%	2	18.2%
Muscle Spasm/Cramps	2	1.2%	2	18.2%
Tremors	2	1.2%	2	18.2%
Hearing Loss/Issues	2	1.2%	1	9.1%
Involuntary Eye Movement	1	0.6%	1	9.1%
Loss of Flexibility	1	0.6%	1	9.1%
Loss of Sensation	1	0.6%	1	9.1%
Nerve Pain	1	0.6%	1	9.1%
Neuropathy	3	1.8%	1	9.1%
Retained Reflexes	1	0.6%	1	9.1%
Vertigo	2	1.2%	1	9.1%
Vision Loss/Issues	3	1.8%	1	9.1%
**Energy-related symptoms**	**28**	**17%**		
Tiredness	18	11.0%	7	63.6%
Fatigue	8	4.9%	6	54.5%
Exhaustion	2	1.2%	1	9.1%
**Genitourinary symptoms**	**1**	**1%**		
Urinary Incontinence	1	0.6%	1	9.1%
**Other symptoms**	**59**	**36%**		
Development of Cardiovascular and Endocrinologic Comorbidities	19	11.6%	9	81.8%
Scoliosis *spine curvature*	18	11.0%	9	81.8%
Foot Abnormalities	5	3.0%	4	36.4%
Back Pain	3	1.8%	2	18.2%
Hyperextension of Limbs	4	2.4%	2	18.2%
Leg Pain	3	1.8%	2	18.2%
Extreme Thirst	1	0.6%	1	9.1%
General Pain/Discomfort	1	0.6%	1	9.1%
Lightheadedness	1	0.6%	1	9.1%
Nausea	1	0.6%	1	9.1%
Sensitivity to Pain	3	1.8%	1	9.1%

Caregivers’ rating of overall FA symptom bothersomeness was a mean of 7.4 and a median of 8 (range 3 to 9).

### Impact burden

Following the exploration of symptom burden, patients were asked to describe the impacts to their lives caused by their symptoms. Caregivers were asked to describe the impacts they observed in the person with FA that they cared for.

Patients reported various impacts (64 impacts in total across nine subdomains) they attributed to their FA (Table 4). At least 70.0% of patients (*n* = 14/20) reported at least one impact related to daily performance and activities, walking, mobility (besides walking), relationships and social impacts, emotional function, and school/work. The impacts most frequently reported by patients included: difficulty with food preparation, difficulty walking, use of walker/cane, use of wheelchair (*n* = 13/20; 65.0% each), social life affected (*n* = 11/20; 55.0%), difficulty dressing (*n* = 10/20; 50.0%), and evolution in changes in walking (*n* = 10/20; 50.0%). All other impacts were reported by 50.0% of patients or less. The following impacts were reported by patients but not by any caregivers: difficulty doing outdoor activities, use of track lift, difficulty bending, difficulty dancing, difficulty jumping, general impacts to mobility, difficulty playing sports, embarrassment, irritability, irritation/annoyance, paranoia, dropping out of school, reduced ability to work outside home, reduced ability to complete school work/studying, difficulty sleeping, and sleep apnea.

**Table 4:** Impact code summary table (Reported by persons with FA, *n* = 20)

Patient-Reported Impacts of Friedreich’s Ataxia	Number Patient Language Expressions within Concept	% of Total Impact Expressions (*n* = 437)	# Patients Contributing to Concept Expression (*n* = 20)	% of Patients Contributing (*n* = 20)
**Changes in daily performance & activities**	**119**	**27%**		
Food Prep	20	4.6%	13	65.0%
Dressing	13	3.0%	10	50.0%
Household Tasks/Chores	9	2.1%	9	45.0%
Daily Activities (not otherwise specified)	10	2.3%	8	40.0%
Needs Adapted Living Space	12	2.7%	8	40.0%
Personal Care/Hygiene	14	3.2%	8	40.0%
Driving	10	2.3%	7	35.0%
Awareness/Caution in Activities	5	1.1%	4	20.0%
Eating/Drinking	9	2.1%	4	20.0%
Lifting/Grabbing/Holding Objects	4	0.9%	4	20.0%
Writing/Typing	1	0.2%	4	20.0%
Lives with Others	3	0.7%	3	15.0%
Errands/Shopping	2	0.5%	2	10.0%
Outdoor Activities	1	0.2%	1	5.0%
Stays Home *during the day*	6	1.4%	1	5.0%
**Impacts to walking**	**123**	**28%**		
Difficulty Walking	25	5.7%	13	65.0%
Use of Walker/Cane	36	8.2%	13	65.0%
Use of Wheelchair *inc. transition to wheelchair*	31	7.1%	13	65.0%
Evolution in Changes in Walking *if not related to walker or wheelchair use*	12	2.7%	10	50.0%
Gait Abnormality	11	2.5%	6	30.0%
Changes to Walking First Noticed	5	1.1%	4	20.0%
Use of Scooter	1	0.2%	1	5.0%
Use of Service Dog	1	0.2%	1	5.0%
Use of Track Lift	1	0.2%	1	5.0%
**Impacts related to mobility (besides walking)**	**44**	**10%**		
Difficulty Running	9	2.1%	8	40.0%
Difficulty Transferring	9	2.1%	6	30.0%
Difficulty with Stairs	5	1.1%	5	25.0%
Difficulty Standing	4	0.9%	4	20.0%
Sports	3	0.7%	3	15.0%
Exercise	2	0.5%	2	10.0%
Fine Motor Skills *not specified otherwise*	3	0.7%	2	10.0%
Mobility *not otherwise specified*	3	0.7%	2	10.0%
Difficulty Bending	3	0.7%	1	5.0%
Difficulty Dancing	1	0.2%	1	5.0%
Difficulty Jumping	1	0.2%	1	5.0%
Riding a Bike	1	0.2%	1	5.0%
**Falls**	**31**	**7%**		
Falls	20	4.6%	8	40.0%
Injuries Due to Falls *broken bones*,* hitting head*,* hip injury*, etc.	5	1.1%	5	25.0%
Frequency of Falls	6	1.4%	3	15.0%
**Relationship and social impacts**	**21**	**5%**		
Social Life *affected*	17	3.9%	11	55.0%
Stigma/Reaction from Others	3	0.7%	2	10.0%
Dating *affected*	1	0.2%	1	5.0%
**Emotional functioning**	**41**	**9%**		
Irritability/Annoyance	13	3.0%	6	30.0%
Fear	5	1.1%	4	20.0%
Anxiety	4	0.9%	3	15.0%
Depression/Sadness	4	0.9%	3	15.0%
Frustration *inc. aggravation*	6	1.4%	3	15.0%
Embarrassment	2	0.5%	2	10.0%
Irritation	2	0.5%	2	10.0%
Anger	1	0.2%	1	5.0%
Paranoia	1	0.2%	1	5.0%
Self-Consciousness/Insecurity	1	0.2%	1	5.0%
Stubbornness	2	0.5%	1	5.0%
**School/work impacts**	**42**	**10%**		
Losing Employment/Employment Opportunities	9	2.1%	6	30.0%
Missing School/Work	6	1.4%	5	25.0%
Does Not Work/Works Less	8	1.8%	5	25.0%
Reduced Ability to Work Outside Home	10	2.3%	4	20.0%
School Work/Studying *reduced ability to complete*	7	1.6%	3	15.0%
Difficulty at School *not otherwise specified*	1	0.2%	1	5.0%
Dropping Out of School	1	0.2%	1	5.0%
**Sleep impacts**	**4**	**1%**		
Sleep Apnea	3	0.7%	3	15.0%
Difficulty Sleeping	1	0.2%	1	5.0%
**Additional impacts**	**12**	**3%**		
Choking inc. aspiration	10	2.3%	7	35.0%
Treatment Burden	2	0.5%	2	10.0%

When asked to rate how their FA impacted their day-to-day life from 1 (no impact at all) to 9 (extremely high impact), patients reported a mean rating of 7.1 and a median of 8 (range 4 to 9).

Patients most often reported the largest burden of FA to be mobility issues (*n* = 4/14 of those asked; 28.6%) and energy-related symptoms (*n* = 4/14; 28.6%). Example quotations are as follows:

*I just feel like every time that I move*,* I have to deal with the FA symptoms… I have to think about my movement.* (Patient 25/female)

*… not even being able to walk but just stand… to be able to transfer by myself.* (Patient 22/female)

Caregivers also reported a variety of impacts they observed as affecting their children (64 in total across nine domains) (Table 5). Ninety to 100% of caregivers (*n* = 10/11) reported observing at least one impact related to daily performance and activities, walking, mobility (besides walking), falls, relationships and social impacts, emotional function, and school/work. The impacts most frequently reported by caregivers included: difficulty with personal care/hygiene (*n* = 10/11; 90.0%), difficulty walking (*n* = 10/11; 90.0%), falls (*n* = 10/11; 90.0%), and social life affected (*n* = 10/11; 90.0%), gait abnormality (*n* = 9/11; 81.8%), family life affected (*n* = 9/11; 81.8%), difficulty dressing (*n* = 8/11; 72.7%), reduced ability to complete school work/studying (*n* = 8/11; 72.7%), difficulty eating/drinking (*n* = 7/11; 63.6%), difficulty lifting/grabbing/holding objects (*n* = 7/11; 63.6%), use of wheelchair (*n* = 7/11; 63.6%), need for adapted living space (*n* = 6/11; 54.5%), use of walker/cane (*n* = 6/11; 54.5%), difficulty running (*n* = 6/11; 54.5%), and missing school/work (*n* = 6/11; 54.5%). All other impacts were reported as observed by 50.0% of caregivers or less.

**Table 5:** Impact code summary table (Reported by caregivers of persons with FA, *n* = 11)

Caregiver-Reported Impacts of Friedreich’s Ataxia	Number Caregiver Language Expressions within Concept	% of Total Impact Expressions (*n* = 504)	# Caregivers Contributing to Concept Expression (*n* = 11)	% of Caregivers Contributing (*n* = 11)
**Changes in daily performance & activities**	146	**29%**		
Personal Care/Hygiene	24	4.8%	10	90.9%
Dressing	28	5.6%	8	72.7%
Eating/Drinking	14	2.8%	7	63.6%
Lifting/Grabbing/Holding Objects	13	2.6%	7	63.6%
Needs Adapted Living Space	8	1.6%	6	54.5%
Daily Activities *not otherwise specified*	9	1.8%	5	45.5%
Awareness/Caution in Activities	11	2.2%	5	45.5%
Driving	7	1.4%	5	45.5%
Food Prep	11	2.2%	5	45.5%
Writing/Typing	9	1.8%	4	36.4%
Other School Activities	3	0.6%	3	27.3%
Household Tasks/Chores	3	0.6%	2	18.2%
Lives with Others	2	0.4%	2	18.2%
Travel	2	0.4%	2	18.2%
Errands/Shopping	1	0.2%	1	9.1%
Stays Home *during the day*	1	0.2%	1	9.1%
**Impacts to walking**	**90**	**18%**		
Difficulty Walking	28	5.6%	10	90.9%
Gait Abnormality	12	2.4%	9	81.8%
Use of Wheelchair inc. transition to wheelchair	16	3.2%	7	63.6%
Use of Walker/Cane	19	3.8%	6	54.5%
Evolution in Changes in Walking if not related to walker or wheelchair use	9	1.8%	5	45.5%
Changes to Walking First Noticed	3	0.6%	2	18.2%
Use of Service Dog	2	0.4%	2	18.2%
Use of Scooter	1	0.2%	1	9.1%
**Impacts related to mobility (besides walking)**	**43**	**9%**		
Difficulty Running	6	1.2%	6	54.5%
Difficulty Standing	6	1.2%	5	45.5%
Difficulty Transferring	14	2.8%	5	45.5%
Fine Motor Skills *not specified otherwise*	5	1.0%	4	36.4%
Riding a Bike	5	1.0%	4	36.4%
Difficulty with Stairs	3	0.6%	3	27.3%
Difficulty Sitting	2	0.4%	1	9.1%
Difficulty Standing Up	1	0.2%	1	9.1%
Exercise	1	0.2%	1	9.1%
**Falls**	**69**	**14%**		
Falls	36	7.1%	10	90.9%
Frequency of Falls	21	4.2%	10	90.9%
Injuries Due to Falls *broken bones*,* hitting head*,* hip injury*, etc.	12	2.4%	4	36.4%
**Relationship and social impacts**	**48**	**10%**		
Social Life *affected*	25	5.0%	10	90.9%
Family Life *affected*	14	2.8%	9	81.8%
Stigma/Reaction from Others	7	1.4%	4	36.4%
Dating *affected*	2	0.4%	2	18.2%
**Emotional functioning**	**48**	**10%**		
Anxiety	11	2.2%	5	45.5%
Self-Consciousness/Insecurity	5	1.0%	5	45.5%
Anger	5	1.0%	4	36.4%
Depression/Sadness	8	1.6%	4	36.4%
Frustration *inc. aggravation*	7	1.4%	4	36.4%
Worry/Concern	5	1.0%	4	36.4%
Fear	2	0.4%	2	18.2%
Stubbornness	2	0.4%	2	18.2%
General Emotional Health Impact	1	0.2%	1	9.1%
Guilt	1	0.2%	1	9.1%
Mood Swings	1	0.2%	1	9.1%
**School/work impacts**	**40**	**8%**		
School Work/Studying *reduced ability to complete*	13	2.6%	8	72.7%
Missing School/Work	13	2.6%	6	54.5%
Does Not Work/Works Less	3	0.6%	2	18.2%
Losing Employment/Employment Opportunities	5	1.0%	2	18.2%
Reduced Ability to Work Outside Home	2	0.4%	2	18.2%
Difficulty at School *not otherwise specified*	1	0.2%	1	9.1%
Difficulty Getting to School	1	0.2%	1	9.1%
Needs to Sit at Work	1	0.2%	1	9.1%
Need for Support at School	1	0.2%	1	9.1%
**Sleep impacts**	**7**	**1%**		
Sleeps Deeply/Late	7	1.4%	3	27.3%
**Additional impacts**	**13**	**3%**		
Change in Life’s Plans	8	1.6%	5	45.5%
Choking *inc. aspiration*	3	0.6%	3	27.3%
Uses Ventilator	2	0.4%	1	9.1%

Caregivers provided a rating on how their child’s FA impacted their child’s day-to-day life from 1 (no impact at all) to 9 (extremely high impact) NRS. The mean rating was 5.8, with a median of 5 (range 3 to 9).

Caregivers also touched upon the impacts they experienced personally. All caregivers (*n* = 11; 100%) reported being affected by their caregiving responsibilities, most commonly in areas of emotional health, social life, and employment. While 81.8% of caregivers were in full-time employment, the amount of time caregivers spent providing care was an average of 55.5 h per week.

### Financial burden

When asked to rate their current financial burden associated with FA on a 1 to 9 NRS, patients reported a mean rating of 6.5, with a median of 7 (range 2 to 9), while caregivers reported a mean rating of 5.3, with a median of 5 (range 1 to 9) (Table 6). Patients explained they chose their rating of financial burden due to not being able to work due to FA, costs associated with their medical care, and costs related to wheelchairs and adaptive devices. Some patients acknowledged that they did not currently experience any financial burden, or that it was minimal to them. Some caregivers experienced a high financial impact burden related to the costs of medical care, adaptive devices, and medications; other caregivers reported a low burden.

**Table 6:** Financial burden rating (Reported by persons with FA and caregivers for persons with FA, *n* = 31)

Rating of Financial Burden Due to FA	Patients *n* = 16*	Caregivers *n* = 7^
On a scale of 1 to 9 where 1 = no burden at all and 9 = extremely high burden, how would you describe your **current financial burden** associated with FA?		
**Mean**	6.5	5.3
**Median [Range]**	7 [2–9]	5 [1–9]
**Example Quotations**	• (2) *… we are waiting for the treatment. I’m not sure if my insurance will cover that… I haven’t had to deal with a lot of medical procedures*,* so at this moment it’s okay… a two.* (Patient 25/female) • (7) *… seven*,* because I do have quite a few specialists and even on insurance*,* like $50*,* every time I go see a specialist*,* it adds up.* (Patient 31/female) • (8) *… eight… the insurance gave me a referral for a walker… It was not what I wanted specifically*,* so I had to pay out of pocket for the one I want.* (Patient 25/female)	• (3) *Insurance and drug trials have paid for most of everything that we’ve done in terms of*,* like all the thousands of dollars that have been spent have largely been covered and very little out of pocket.* (CG of 13-year-old) • (4) *Mostly it’s just because some of her medication is not covered… We pay for her physical therapy. Her insurance pays for so many visits*,* and then out of pocket*,* we pay for the rest.* (CG of 27-year-old) • (9) … *you got to think about all the assistive devices that he needs that a lot of times it’s more burdened to go through insurance.* (CG of 12-year-old)

* Four patients (20.0%) were not asked or did not answer this question

^ Four caregivers (36.4%) were not asked or did not answer this question

Both patients and caregivers described the increased burden of FA-related costs such as medical expenses, purchases related to home adaptations/accessibility equipment, specialized vehicles, walker/brace costs, wheelchair costs and travels costs. The burden of other costs for patients included inability to pay back student loans and lower paying jobs. Caregivers reported days/time off work due to their children’s FA. Both patients and caregivers often detailed the difficulty they had obtaining insurance coverage for diagnostic tests, follow up visits, and necessary equipment.

### Survey results

All patients (*n* = 20) completed the EQ-5D-3 L and FA-ADL in their pre-interview survey, and both patients and caregivers (*n* = 11) completed the WPAI-SHP about the impact of caregiving on their work and activities. On the EQ-5D-3 L, on average, patients reported their current health as 61.7 on the EQ VAS (health thermometer) from a possible range of 0 to 100 (0 being worse; the actual range was 5 to 95). This is nearly 20 points lower than the US population norm of 80. The calculated EQ-5D index or utility score for the overall population was 0.40 with a range of -0.01 to 0.73 [12]. This is considerably lower than the general US population of 0.87 indicating that this FA population reported poorer health status than the normed US population [12].

WPAI-SHP results showed that more than half of the patients (*n* = 11; 55%) are currently employed, and they reported working an average of 24.3 h during the past seven days. Most reported not missing work in the past seven days due to their FA (*n* = 7; 63.6%) or any other reasons (*n* = 8; 72.7%). Those who missed work reported missing an average of 3.4 h of work due to health problems with FA, and 2.6 h due to any other reason in the past seven days. On a scale of 0 to 10, patients rated that FA affected their productivity at work as an average of 3.8 and rated that FA affected their ability to do regular daily activities other than work as an average of 4.0. Average impairment percentages ranged from 13.0% (absenteeism) to 47.8% (work productivity loss).

Nearly all caregivers (*n* = 10; 90.9%) reported that they are currently employed, and they worked an average of 37.4 h the last seven days. Most caregivers reported not missing their work in the past seven days due to caring for the person with FA (*n* = 7; 70%) or any other reasons (*n* = 7; 70%).The caregivers who missed work missed an average of 2.0 h of work due to caring for the person with FA and 1.1 h of work due to any other reason in the past seven days;. On a scale of 0 to 10, caregivers rated the effect of the person’s FA on their productivity at work as an average of 4.3, and they rated the effect of the person’s FA on their ability to do regular daily activities other than work as an average of 5.2. Average impairment percentages ranged from 3.9% (absenteeism) to 51.8% (activity impairment).

On the FA-ADL, mean item scores (on a possible range of 0 to 4, with 4 being worse) were: speech (2.00), ability to swallow (1.75), ability to cut food (2.25), ability to self dress (2.10), perform personal hygiene activities (2.05), often fall (3.10), ability to walk (3.45), quality of sitting position (1.80), and bladder function (1.65).

## Discussion

The concept of patient burden includes a holistic view of patients’ experience and their ability to tolerate the various aspects of their condition and treatment. Literature supports burden as a multidimensional concept [13], encompassing symptoms (that may be severe, bothersome, and/or frequent), which in turn, impact all aspects of patient daily life, functional outcomes and QOL. Burden can also encompass aspects of treatment that add to burden, including inconveniences related to duration of treatment, location of treatment, and mode of administration. This study explored various aspects of patient burden with FA, including the burden of the diagnostic process, the burden of FA symptoms and impacts, the financial burden of managing FA, and negative experiences with treatment for FA, and contributes further understanding of this to literature. This study’s unique contribution to the FA literature is that it included the perspectives of both patients with FA and caregivers of adults with FA, collected complementary quantitative and qualitative data, and explored various aspects of burden.

Most patients indicated it took them two to three years to receive a diagnosis, while most caregivers reported it took six months to one year for their child to receive an FA diagnosis; this is not unexpected given caregivers were caring for different patients than those interviewed. In addition, diagnosis with FA often occurred only after participants saw at least four HCPs, confirming that the process of diagnosing FA is burdensome to both persons with FA and their caregivers, who may be responsible for making appointments with HCPs, coordinating communication between HCPs, and transporting patients to appointments. Research on the FA diagnosis process from the perspective of patients and caregivers is lacking, but our results support findings from qualitative research in other rare diseases [14].

The most frequently reported symptoms for patients and caregivers were impaired speech, muscle coordination decrease, loss of balance, fatigue, tiredness, development of cardiovascular and scoliosis. Endocrinologic comorbidities were also mentioned. These results are in line with findings from other studies [1, 3]. Symptom reports were extremely varied and some were reported by very small proportions of patients/caregivers, highlighting the heterogeneous experience of patients and caregivers who are living with FA. The most frequently reported impacts for patients and caregivers were difficulty with food preparation, difficulty with personal care/hygiene, difficulty walking, falls, use of walker/cane, use of wheelchair, and social life affected. Though both patients and caregivers reported a large number of impacts and included impacts the other group did not report, caregivers generally reported impacts at greater frequencies than patients (e.g., personal care/hygiene and falls). These differences in reporting may be due to caregivers noticing symptoms and impacts that patients experience but have become accustomed to. It is important to remember the caregivers in this study were not caregivers for the patients in the study; some difference in reported symptoms and impacts is expected, so the content between patient reported symptoms and impacts cannot be directly compared to those reported by the caregivers.

Our results on symptom and impact burden mostly mirror those of Seabury et al. [15], whose study examined the prevalence and relative importance of FA symptoms but included what we categorize as impacts as symptoms. They found the most prevalent symptoms to be impaired coordination, fatigue, and lower extremity weakness, and the most prevalent impacts (also called symptoms in their study) to be inability to do activities and limitations with mobility and walking. In addition to examining symptom/impact prevalence, they also assessed importance of symptoms/impacts and found that the most common symptoms and impacts were also reported by participants to be the most burdensome.

Financial burden associated with FA was most often related to medical care, medications, wheelchairs, and other adaptive devices. Our study adds to the literature in showing that caregivers also report financial burden associated with caring for someone with FA, though they rated their financial burden near the midpoint of the 1 to 9 scale (5.3), compared to the slightly higher rating by patients (6.5). This study also showed that patients with FA reported a higher financial burden than caregivers, which may be due to the impact of not working full-time or being unemployed.

PRO results from the pre-interview survey underscore the burden described by patients and caregivers during their interviews. The EQ-5D-3 L results categorize the patient sample as having much worse health than the general population, while the patient sample was near the midpoint of the condition-specific FA-ADL. This exemplifies that condition-specific PROs may be better at assessing health of patients as they are constructed to focus on concepts important to assess for the condition specifically, as opposed to general function or health status.

There were several limitations to this study. The generalizability of study results may be limited due to convenience sampling, as study participants who were recruited through FA patient groups and social media may be more engaged with the FA community than the general population of people with FA. Three quarters of patients with FA were female, which may not be a true representation of the general population of people with FA. In addition, the caregiver sample was limited due to challenges in recruitment. The interview guides asked for symptoms and impacts but did not probe further for typical ones not mentioned by patients and caregivers on their own, which may have resulted in incomplete results of symptom and impact experience. The full content of the interview guide may not have been presented in all cases due to lack of time and low stamina of participants. As a result, prevalence of symptoms and impacts may be lower than if the interview guide was designed differently. One additional limitation is that saturation of concept was not achieved; however, it should be noted that the descriptions of the concepts brought up in the last group of interviews were highly detailed and individual to persons with FA and caregiver preferred terminology, making them quite granular in comparison to other concepts reported.

Future research in this area is welcomed and could focus on the different patient and caregiver burden across alternative patient subgroups, such as early vs. late onset and ambulant vs. non ambulant patients. Understanding of these findings as consistent globally would also be of value.

## Conclusions

This study demonstrates the tremendous burden that FA has on both patients and caregivers and the meaningful impact it has on many aspects of their lives, including physical, social, emotional, and financial. Awareness of this burden may assist with the identification of unmet needs and support the patient through improved care pathways. Continued treatment evolution and expanded support to FA patients and their caregivers are important to reducing the burden of FA.

## Data Availability

All data generated or analyzed during this study are included in this published article [and its supplementary information files].

## Abbreviations

EU: European Union
FA-ADL: Friedreich Ataxia – Activities of Daily Living
FA-HI: Friedreich's Ataxia-Health Index
FA: Friedreich ataxia
FARA: Friedreich’s Ataxia Research Alliance
HCP: Healthcare provider
ICA: Inter-coder agreement
NRS: Numerical rating scale
PRO: Patient-reported outcome
QAP: Qualitative analysis plan
QOL: Quality of life
US: United States
VAS: Visual analogue scale
WPAI-SHP: Work Productivity and Activity Impairment
